# The ward round: friend or foe in postgraduate training? A grounded theory study of residents’ perspectives

**DOI:** 10.1080/10872981.2022.2101180

**Published:** 2022-07-18

**Authors:** Mariam Noorani

**Affiliations:** Consultant Paediatrician and Senior Instructor Department of Paediatrics, Aga Khan University, Dar Es Salaam, Tanzania

**Keywords:** Ward round, postgraduate training, medical education, grounded theory

## Abstract

The ward round has traditionally been a learning activity in medical education. Apart from education, ward rounds have multiple roles including patient care and communication. Some studies have described the ward round as an ideal place to learn patient management while others reported that little learning happens on rounds due to lack of time and patient volume. This study aimed to develop a deeper understanding of ward round learning from the perspective of postgraduate trainees. A constructivist grounded theory approach was used and data was collected during focus group discussions. Data were analyzed by initial coding, then grouped into focused codes and development of a theoretical framework by the process of constant comparison. Six categories evolved which contributed to the framework. Postgraduate trainees perceive the ward round as an important space where they use different learning activities to acquire knowledge, attitude and skills required of a specialist doctor. They progress from novices to experts under supervision of faculty who lead ward rounds. The round can achieve its full learning potential if planned and organized well but can become a missed opportunity if the learning environment is unfriendly. Patient- and learner-related barriers exist that hinder ward round learning. The framework explains how ward round learning occurs in postgraduate medical education from a trainee perspective. The findings can guide interventions to improve the learning experience. Studies comparing perspectives of teachers to those of learners are needed to further understand the complex learning milieu of the ward round.

## Introduction

The ward round has played an integral role in the training of doctors for many generations. Existing literature attributes the principles and practice of the ward round to Sir William Osler who was a strong advocate for bedside teaching [1].

In recent years, medical education has changed from the traditional apprenticeship model to a more evidenced based approach to teaching and learning [2]. Advances in technology and educational approaches have changed how students learn in this era of digital education. The ward round itself has undergone major transitions. The traditional ward round was described as a parade where the senior physician was followed by junior doctors and students as they moved from patient to patient [3]. Modern day ward rounds have taken many forms including conference room rounds, family-centered rounds and multi-disciplinary ward rounds [4].

Apart from teaching and learning, the ward round has multiple other roles including patient care, communication of care plans, reviewing of diagnostic test results and completion of documentation [5]. These other tasks can substantially reduce teaching time. In addition, faculty members who are responsible for teaching usually have other competing responsibilities including research, administrative duties and clinics. This workload reduces time available for ward round teaching [6].

Considering the complex nature and the changing face of the ward round, it is important that the learning that occurs during rounds and its contribution to the training of doctors be re-examined. Studies that have evaluated the educational value of rounds for post graduate trainees have shown conflicting results. Some trainees described the ward as an ideal place to learn patient management while others expressed that very little learning occurs during rounds [7–9]. Barriers to learning on ward rounds and various ways to improve learning have been elaborated [4,10]. However, few studies have explored fully the process of ward round learning in postgraduate training; what is learnt on rounds, how learning is achieved and the roles of ward round participants.

Additionally, existing literature on ward round learning comes from high-income settings where health care and educational systems differ greatly compared to low resource Sub Saharan African settings. To fill the existing gaps, a qualitative study using constructivist grounded theory was conducted to develop an in-depth understanding of learning during ward rounds from the perspective of postgraduate trainees.

## Methods

### Study design

This qualitative study was conducted using grounded theory methodology based on the research question which aimed to develop an in-depth understanding of how learning is achieved during ward rounds. With an epistemological stance of constructivism which holds that knowledge is actively constructed and co-created as the product of human interactions; a constructivist grounded theory approach was used [11].

### Study population and site

The study population was postgraduate trainees [residents] from 2^nd^ to 4^th^ year who were enrolled into Masters of Medicine programs in Dar es Salaam – Tanzania.

Participants were from one public university [Muhimbili University of Health and Allied Sciences – MUHAS] and one private university [The Aga Khan University – AKU] to ensure that the data collected were rich and diverse and the theory generated could be applied to a wide range of similar settings.

MUHAS residents undertake their training at the country’s largest public teaching and referral hospital; the Muhimbili National Hospital (MNH). Ward rounds in this setting are led by both physicians employed by MNH as well as faculty members from MUHAS. The MNH physicians have a predominantly service mandate while the MUHAS faculty members have a predominantly teaching mandate.

Residents of the AKU undertake their training at the Aga Khan Hospital (AKH) which is the country’s largest private health care and referral system. Ward rounds in this setting are led by the faculty members of the AKU who are the same as those working at the AKH, hence they have both a teaching and a service mandate.

### Sample size and sampling strategy

The initial strategy used was purposive sampling; all residents from 2^nd^ to 4^th^ year who were involved in ward rounds during their training were informed about the study through their student leadership and invited to participate. Fifteen residents responded to the initial invitation and enrolled into the study. Subsequent sampling was theoretical sampling where data was collected from sources that would facilitate further development and refining of the theory [12]. Residents from surgical disciplines like otolaryngology who did not respond to the initial call were contacted directly to participate in order to get data from all possible specialties. The final number of residents who participated was 20 with the following distribution: paediatrics – 6, obstetrics – 1, internal medicine – 5, family medicine – 5, surgical specialties – 3. There was an equal number of male and female residents and 11 of them were in their 2^nd^ year of training while the rest were in their senior years of residency.

### Data collection

Data was collected during focus group discussions [FGD] between April and August 2021. Using focus groups, a range of different view-points, ideas, feelings and differences in perspectives between individuals can be illuminated [13]. Four heterogenous FGDs were conducted with between 3 and 6 residents in each group from different years, specialties and universities. During the third FGD, most of the discussion was around similar thematic areas that were discussed in the previous FGDs. A fourth FGD was conducted to capture any new issues of interest after which it was concluded that a point of data saturation had been reached and the data were adequate to answer the research question.

The groups were moderated by the primary investigator using a topic guide. Each FGD lasted between 40 and 50 minutes and discussions were recorded on a voice recorder. A colleague who was not a study investigator attended as an observer to document the interactions and recorded her perspectives of the data including non-verbal cues [14]. The primary investigator and observer met at the end of each FGD to share thoughts and ideas that were used to plan for the subsequent FGD.

### Topic guide

This was developed by the primary investigator and is available as a supplementary file. The questions were open ended and had three main areas of focus which moved from general to more specific as recommended by Krueger [15]. The first was to understand the format and structure of rounds, second was to explore their perceptions of what they learn, how they learn and the roles played by faculty and learners. The third was to identify barriers and facilitators to learning.

During the first FGD, it was noted by the observer that the study participants were giving general responses without specific examples; subsequently, the FGD topic guide was modified to ask participants to highlight specific examples from their learning experiences.

### Data analysis

This was done as an iterative process as data collection proceeded. The recordings were transcribed using an online speech to text converter and transcripts were reviewed manually to verify accuracy with audio recordings. Initial analysis involved line by line coding as described by Charmaz [11]. A code was assigned to each line using gerunds; verbs ending in “ing” that act as nouns; derived from the actual words or feelings of participants. Use of gerunds helped to define what is happening in the data and identify the theoretical direction in the code while constantly remaining close to the data [16].

Subsequent coding was focused coding where relationships between initial codes were explored and similar codes grouped together. Throughout analysis, the principle of constant comparison was used [12] where emerging codes were compared with previous examples from the same FGD as well as previous discussions for similarities and differences.

Final coding was theoretical coding which specified relationships between focused codes and helped tell a coherent analytic story [11]. These formed the six core categories which contributed to the final theoretical framework. Diagrams were used to show a visual representation of emerging concepts while tables were used to record and compare codes in a word processing software.

In constructivist grounded theory, the researcher is an important part of the process and researcher bias can emerge [17]. To ensure that participant’s voices contributed to the theory rather than the author’s perceptions, the author constantly engaged in researcher reflexivity which is the process associated with researchers’ self-awareness; of how they impact and transform the research they undertake [18]. During analysis; memo writing was used to document insights and reactions. Memos were revisited to facilitate interpretation of the data and ensure that the emerging codes and categories are grounded in the data and not the author’s own sentiments.

### Ethical considerations

Ethical approval was obtained from the ethics review committees of both universities where participants were recruited [AKU/2021/025/fb, DA 282/298/01/C/10] and all participants gave written informed consent. Prior to starting each FGD, participants were requested to maintain confidentiality of the discussions and all data was stored in a password protected laptop. Data coding and analysis was done anonymously without identifying individuals and any identifying information was removed from the quotes.

## Findings

Six conceptual categories evolved from data analysis and were used to construct the following theoretical framework conceptualizing ward round learning in post-graduate medical education:

Postgraduate trainees perceive the ward round as an important space where they use different learning activities to acquire knowledge, attitude and skills required of a specialist doctor. They progress from novices to experts under supervision of faculty who lead ward rounds. The round can achieve its full learning potential if planned and organized well but can become a missed opportunity if the learning environment is unfriendly. Patient- and learner-related barriers exist that hinder ward round learning.

**Figure 1.**
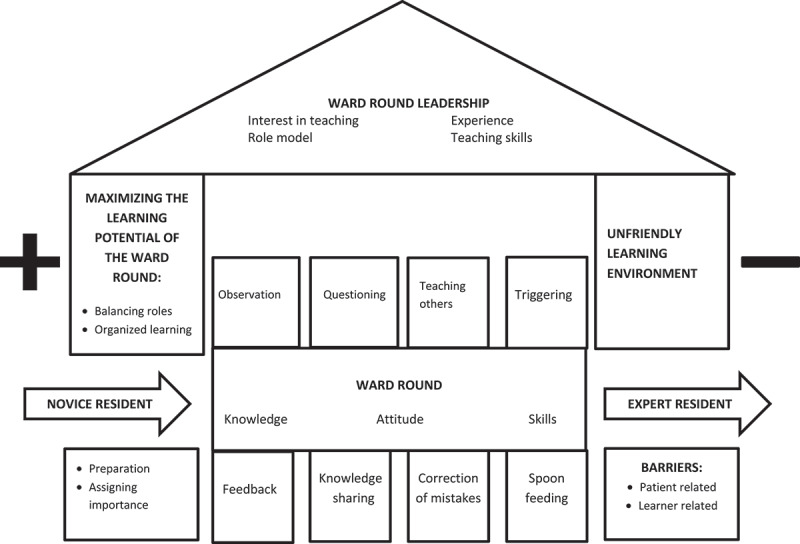
Diagram conceptualizing ward round learning in postgraduate medical education.

An illustration of this is depicted in Figure 1.

The categories and focused codes that form each category are summarized in Table 1. These are further explained with relevant quotes from participants.

**Table 1.** Category development from focused codes.

Theoretical category	Focused codes
From novice to expert – learning knowledge, attitude and skills to become a specialist	Learning and applying higher order knowledge and skills Gaining mastery of non-technical skills Linking up literature and patient care Progression from supervised to independent practice
Teaching and learning activities on the ward round	Making mistakes and being corrected Learning by observation Questioning as a way to learn Constructive feedback Knowledge sharing Spoon feeding Teaching others Triggering
The critical role of ward round leadership	Interest of faculty in teaching Need for teaching skills Faculty as a role-model The power of experience
Maximizing the learning potential of the round	Balancing ward round roles Organized learning on the ward round Adequate preparation for the ward round Assigning importance to ward round learning
An unfriendly ward round learning environment – a missed opportunity	Fear and anxiety during roundsHopelessness and reduced self-esteem Harassment during rounds
Barriers to learning on ward rounds	Different cadres of learners Large number of trainees Busy rounds Patient preferences

### Category 1: from novice to expert – learning knowledge, attitude and skills to become a specialist

The ward round was described as a place where residents learn what is required to become a specialist. They acquire higher order knowledge and skills such as clinical reasoning and shared decision-making and apply these to patient care.

*“They [faculty] actually make you rethink about the decisions you made and they will always ask you, why did you do this and to what logic reasoning did you use to come up with this decision”*

*“We learn how to convey information in a manner that the patient will understand, so that they’ll make an informed decision about their plan, accepting the management or choosing an alternative.”*

They also learn non-technical skills including communication, documentation and leadership which are not traditionally taught in classrooms but are important for patient care.

*“You are with a specialist and you see them responding to the question that had been asked, giving the bad news, giving feedback to the patient. Yeah, so this is the kind of thing that when you are alone now, when these questions are coming to you, you will answer them”*

The participants elaborated how faculty guides them on linking up what they read in literature with actual patient care on the ward rounds.

*“You’re going to check out what are the current guidelines in managing this patient, and do you think that what we have implemented is with the guidelines or not, let’s sit and discuss whether we need to make any changes to the treatment plan.”*

The progression from supervised to independent clinical practice also happens on ward rounds.

*“The first time they would be doing it, they would tell you, you have to do this and that, then later on they will allow you, they let you do it so that’s how you get the skill”*

### Category 2: teaching and learning activities on the ward round

The question of how residents learn on ward rounds was answered with a multitude of activities. Discussions amongst residents and their faculty provided a platform to share knowledge and stimulate further reading but this only happened if faculty were accommodative and facilitated such discussions.

Faculty commonly used questioning as a way to gauge residents’ knowledge.

*“When you are asked a question, it tells you a lot about how much you know and how much you don’t know”*

The ward round was considered a safe space to make mistakes and be corrected without compromising patient care.

*“So if you have a plan, and then your specialist sees your plan, they correct your plan from what their experience has taught them”*

The power of visual learning was clearly articulated where residents observe clinical and social skills of their faculty then emulate and apply the same.

*“So I will respond to this question, as I have seen my specialist respond to these questions. We are not directly taught about this.”*

*“The skills that you read in the book, sometimes it’s hard to perform them until you see someone performing that skill.”*

Getting feedback during rounds was cited as an important way of learning. Various situations were elaborated where feedback was provided including physical examinations, management planning and communication skills.

*“When you get a feedback either positive or negative, but if it’s constructive, it gives you something to learn.”*

Residents were involved in the teaching of medical students and interns during rounds. They described teaching as a way they identify their own knowledge gaps.

*“For me the better way of learning was to teach, it tells you about how much you know, so I think when you teach, it makes you remember a lot of things.”*

During first year of training, didactic teaching was common and was described as spoon-feeding where faculty explained concepts from one patient to the next. Residents also described triggering as a way to learn.

*“Whatever they are saying is very little compared to what we need to gain so they trigger us and we go and read then we will come back ourselves to the patients.”*

### Category 3: the critical role of ward round leadership

An overarching recurrent theme throughout all FGDs was the critical role of the person leading ward rounds. Residents viewed the leader as a pillar that makes the round a learning activity and they had high expectations of faculty leading rounds.

*“Some people who are training us have no knowledge of how to train us.”* This sentiment echoed throughout the discussions. The expectation was that specialists leading rounds should have teaching skills and be able to teach different types of learners. Being an expert in their field does not qualify them as a teacher.

Residents respected the role of experience in teaching and learning. Even though they may have more knowledge as trainees, they recognized that experience of faculty plays a key role in learning more so when dealing with rare conditions.

*“I believe residents at this point in time read a lot, but we just read everything, we don’t have the experience. So they [faculty] come in and they probe the high yield things based on that patient.”*

*“In medicine, seniority plays a part in the learning process, you find a case that none of you guys have seen, but as long as you have a senior specialist who has come across that case before, they will tell you, by experience we do this and this and it works.”*

An important realization was that various cadres of specialists lead rounds and not all are academic faculty. Some are hospital employees who do not have a teaching mandate and no interest in teaching.

*“If those academicians are there its very different compared to when these specialists who are employed by the hospital are there. When an academician attends the rounds teaching occurs.”’*

### Category 4: maximizing the learning potential of the round

Ward rounds have multiple competing roles and there was evident frustration that these were not balanced with clinical care taking priority over teaching and learning. This was even more evident during the COVID-19 pandemic surge where they had to deal with large numbers of patients.

*‘It’s not okay to go for a whole month doing business round at the teaching hospital.’*

A universal agreement was that learning on the ward round must have a structure and be planned beforehand. Impromptu learning without clear goals becomes uninteresting, overwhelming and has no benefit.

*“You see like 30 patients with 20 diagnosis and you’re trying to make me learn from each and every patient, by the end of the day, I would have panicked maybe, because I feel like I don’t know anything now.”*

Planning learning objectives and assigning learning tasks beforehand were suggested as ways to organize learning.

Preparation for ward rounds was cited an important pre-requisite to facilitate learning. Poorly prepared rounds resulted in focus being shifted to tasks like tracing results and filling documents. However, residents admitted that how well preparation is done depends on the faculty member who will lead the round.

*“We know this faculty is very strict, she wants everything to be done perfectly, you need to prepare yourself.”*

It was interesting that residents sometimes did not attend ward rounds and instead spent time doing self-study. This is because ward round participation does not carry weight towards assessments which are always in the form of written exams. They suggested that including ward round activities as part of assessment would improve participation.

### Category 5: an unfriendly ward round learning environment – a missed opportunity

The need for a friendly environment during rounds was a consistent theme echoed by residents from different departments and years of study. They gave vivid examples of how faculty make the round a place of fear, anxiety and negativity; ultimately resulting in a missed learning opportunity.

*“Even if it is the time for this specialist to teach me I don’t understand because I am really worried this person is so harsh, harsh on me I’m really worried .I wish he could finish and off he goes.”*

*“Sometimes the learning process is not a learning process. It’s a place that they make you more inferior, they make you lose hope, and they make you not participate.”*

*“Just from the beginning of the round, you’re being accused of this and that, then maybe at times you are chased from the round.”*

### Category 6: barriers to learning on ward rounds

Ward rounds that have many trainees including undergraduate, postgraduate and interns resulted in compromised postgraduate-level learning.

*“You realize that the teaching on the same patient is actually quite repetitive or prolonged because they have to cater for all academic audiences.”*

Ward rounds that had a large number of patients became overwhelming. Even though the patients had conditions of learning interest, the time limitation and need to complete patient care requirements hindered learning.

## Discussion

“Medicine is learned by the bedside and not in the classroom.” These words of wisdom said by Sir William Osler over a century ago still resonate in the corridors of teaching hospitals today where bedside learning is frequently embedded in ward rounds. The modern day ward round has been the subject of many studies which have examined individual components of rounds such as roles [19], educational value [8] and teaching and learning strategies [20]. This study is the first to propose a framework which gives an in-depth understanding of learning during rounds from a trainee perspective and can be used as lens to look at this important learning activity in postgraduate training.

Residents in this study described the ward round as a place to achieve diverse learning including acquisition of non-technical skills. They readily acknowledged the round as the best place for learning despite its multiple other roles including patient care. Many studies in the existing literature have reported low educational value of rounds because of barriers such as lack of time, increasing patient numbers and lack of team consistency. Claridge reported that less than 20% of learning occurs on rounds and only about 9% of the round is dedicated to teaching [8]. A study from Pakistan reported that residents desired more from their rounds especially focus on communication skills, counseling and medical ethics [9]. Similarly, an ethnographic study observing ward rounds highlighted that they were mainly focused on technical content with little focus on other areas of professionalism [21].

The differences in perception of the educational value of ward rounds between the present study and existing literature could be explained by the differences in methods used. Previous studies used questionnaires which limit responses and an in-depth understanding cannot be obtained. In the ethnographic approach, interactions are observed rather than reported. Observation may not be able to identify implicit learning that occurs as part of social interactions in the work place as explained by the social cognitive theory where individual factors, environmental factors and behaviour interact to enable learning [22]. The present study, being of a qualitative nature, has been able to explore different learning approaches.

Ward round leadership was identified as the critical driver for learning on rounds. Faculty leading rounds were expected to have an interest in teaching and the skills to teach learners at postgraduate level. Lack of teaching skills has been highlighted previously as a barrier to ward round learning [10] and various studies have recommended faculty development as a strategy to promote teaching on bedside rounds [23,24].

A variety of teaching and learning activities were described in this study. These varied depending on the seniority of the trainee, the type of knowledge or skill being learnt and the faculty who was teaching. Feedback was considered an important learning activity and trainees desired constructive feedback even for simple tasks. Educational activities including feedback were reported in a study involving paediatric residents [25] where feedback was noted to be scarce and only provided when something had gone wrong.

Teaching of junior colleagues has been described as near – peer teaching and it helped residents recognize how much they know and areas where they have gaps. This is in contrast to a previous study where senior residents who were teaching felt there was not much learning for them and it reduced their time on the round [26].

Residents recognized that ward rounds have multiple roles and education is just one of those roles. The responsibility of balancing the various roles fell on the shoulders of faculty leading the round. Some suggestions on how to maximise the learning included planning ward round learning objectives and focusing on a particular knowledge or skill strength. Various tools have been reported to promote ward round learning including the STIC [Set, Target, Inspect and Close] framework and tool [27], the one-minute preceptor and SNAPPS models [28].

Training at residency level is presumed to be self-driven with trainees being in charge of their own learning. It was interesting that ward rounds may not be given priority because they do not contribute to assessments. A valid suggestion was that scoring ward round participation would improve resident engagement and learning. This is in keeping with behavioural learning theories that describe learning by use of external stimuli [29].

The ward round learning environment is a key dimension that contributes to successful rounds. In a systematic review, “climate related factors” were described as barriers to learning and included the psychological atmosphere [30]. Fear, anxiety and learner humiliation on rounds have been described in studies involving undergraduate students [31]. At postgraduate level, it is uncommon to have the kind of harassment and negative relationships that the present study has found. This is concerning because such events can have major negative psychological impact on trainees.

Reasons for the unfriendly environment were not explored; however, this may be due to the way in which medical education has always been imparted in this setting. When faculty were trainees, they may have undergone the same humiliation and harassment, hence it perpetuates across generations. This brings to light elements of the hidden curriculum that need further exploration in this setting.

Having a large number of trainees and different cadres of learners were identified as barriers. The quality of teaching for residents in such rounds is compromised and this has also been perceived by teachers on rounds [10]. Patient-related barriers have been identified in multiple studies [6,8] and similar to my findings, having a large number of patients is an obstacle to teaching and learning since clinical care takes priority. However, from the perspective of the study participants, this should not be an excuse for not teaching and faculty has to take the lead to balance the roles.

The barriers highlighted in the ward round learning environment can be collectively viewed and explored using the “avenues” approach to the clinical learning environment (CLE) [32]. This conceptual model approaches the CLE using six different avenues: architectural, digital, diversity and inclusion, education, psychological and sociocultural. Using such a conceptual model can help to organize and stratify the key challenges and subsequently identify strategies for improvement using existing resources.

The unfriendly learning environment can be viewed through the psychological avenue of the CLE [33]. For optimal learning, psychological safety of trainees must be established and sustained and the trainees should be able to speak up against negative behaviours without any personal or professional consequences. The large number of trainees and its impact on learning can be viewed using the avenue of diversity and inclusion [34] while the pressure of patient volume, time and other competing clinical priorities can be approached through the avenue of education [35]. Strategies to improve ward round learning such as faculty development to improve teaching skills could have an impact on multiple avenues of the CLE. Similarly, planning learning objectives before rounds and assigning specific learning tasks to trainees could positively influence the education avenue as well as the psychological avenue.

## Limitations

This study was conducted in an East African country where health and medical education systems are different from other parts of the world. However, the author hopes that the results will be a valuable resource for medical education systems in low- and middle-income countries where there are large numbers of trainees in resource limited health-care systems. Residents from both public and private universities were recruited to give more transferrable findings.

## Conclusion and recommendations

The findings have described how postgraduate trainees achieve learning in the complex milieu of the ward rounds including the facilitators and the barriers to learning. The framework developed from this study can be applied in different settings to identify context-specific areas of intervention to promote ward round learning. This study was a qualitative one that focused on residents; determining the perspective of faculty and comparing this to learner perspectives will give richer data that can better explain learning on the ward round.

Although the ward round is a rich learning environment, this potential can only be fully tapped if faculty who lead rounds recognize their critical role and take specific steps to strengthen the learning including planning and preparing for rounds and ensuring a safe and friendly learning space. This will ensure that the ward round retains its important role in postgraduate training and the legacy of Sir William Osler lives on.

## FGD guide

Please introduce yourselves, your university, your department and your year of study

The ward round has been considered an important part of medical education for a long time now. With time, various different types of ward rounds have emerged.

1. Can you tell me what kind of rounds you engage in during your training?
2. How long are the ward rounds
3. Who usually leads ward rounds
4. How does the ward round contribute to your learning?
  (a) What kind of knowledge is acquired
  (b) What types of clinical skills are learnt
  (c) What about non-technical skills like communication/documentation/leadership
5. What role does the faculty play during your ward rounds?
  (a) How do your faculty enable learning on the ward rounds?
6. What kind of activities do you think enable learning and development on rounds?
  (a) What makes learning on ward rounds more enjoyable?
  (b) What hinders learning during rounds?
7. How do you prepare for the ward rounds?
  (a) What kinds of learning activities/tasks emerge as a result of the ward round?

What are your suggestions on how learning can be improved on the ward round?
